# The Sarcoma-Specific Instrument to Longitudinally Assess Health-Related Outcomes of the Routine Care Cycle

**DOI:** 10.3390/diagnostics13061206

**Published:** 2023-03-22

**Authors:** Nasian Mosku, Philip Heesen, Salome Christen, Mario F. Scaglioni, Beata Bode, Gabriela Studer, Bruno Fuchs

**Affiliations:** 1 Department of Plastic & Reconstructive Surgery, University of Grenoble, 38000 Grenoble, France; 2 Department of Plastic & Reconstructive Surgery, University of Zurich, 8091 Zurich, Switzerland; 3 Department of Health Sciences and Medicine, University of Lucerne, 6000 Lucerne, Switzerlandmario.scaglioni@luks.ch (M.F.S.);; 4 University Teaching Hospital LUKS Lucerne Sarcoma Surgery, University of Lucerne, 6000 Lucerne, Switzerland; 5 Patho Enge, University of Zurich, 8000 Zurich, Switzerland; 6 Kantonsspital Winterthur (KSW), 8400 Winterthur, Switzerland; 7 University Hospital Zurich (USZ), 8000 Zurich, Switzerland

**Keywords:** sarcoma-specific HRQoL instrument (health-related quality of life), PROMs (patient reported outcome measurements), IELAS-RWTD/E (interoperable electronic longitudinal absolute structured real-world-time data/evidence), VBHC (value-based healthcare)

## Abstract

Patient-based health related quality of life (HRQoL) measurements are associated with an improvement in quality of care and outcomes. For a complex disease such as sarcoma, there is no disease-specific questionnaire available which covers all clinically relevant dimensions. Herein, we report on the development of an electronically implemented, sarcoma-specific instrument to assess health-related outcomes, which encompasses a combination of generic questionnaires tailored to the respective disease and treatment status covering the entire longitudinal care cycle. An interoperable digital platform was designed to provide a node between patients and physicians and to integrate the sarcoma-specific HRQoL instrument with patient and physician-based quality indicators to allow longitudinal structured real-world-time data evidence analytics. This approach enables the prediction modeling of disease, and by attributing cost tags to quality indicators, treatment effectiveness for a given disease will be directly correlated with financial expenses, which may ultimately lead to a more sustainable healthcare system.

## 1. Why Do We Need Patient Reported Outcome Measures (PROMs)?

Healthcare costs are constantly rising and impose great challenges [1]. Our healthcare system today is largely ignorant of incorporating treatment effectiveness and outcomes, and rising healthcare costs are leading us toward a wasteful and unsustainable trend [2]. 

Improving value in healthcare is meant to benefit patients, payers, providers and suppliers while increasing the economic sustainability of our healthcare system [3]. Therefore, there is a great need to improve patient-centered care [2] and to possibly redesign a novel healthcare ecosystem with particular focus on shared value [4]. Porter defined shared value as a multidimensional relationship between health outcomes and costs incurred to deliver these outcomes [3,5]. Obviously, there are differences in perceptions of value among patients and between patients and providers [6]. The definition of shared value in healthcare includes the clinical metrics as defined by the physicians as well as by the patients’ voice as assessed by the quality of care. Considering the upcoming healthcare transition as projected for the next decade [7], the definition of quality of care becomes pivotal because our current fee-for-service model will be replaced by a value-based on quality model. In such a model, PROMs are measures used to assess patients’ health or quality of life and represent an integral part of patient-defined quality of care. They were introduced to assess treatment effectiveness and improve outcomes, and are meanwhile a pivotal part of value definition [8,9,10,11,12,13,14]. There are currently numerous PROMs to assess health-related quality of life (HRQoL). Specifically with regard to cancer, they were shown to be prognostic tools such as for outcome, for example in breast cancer, multiple myeloma, colon and lung cancers [15,16,17,18]. PROMs are generally used at the aggregate level for audit and benchmarking, real world evidence generation and as an input or predicted output for clinical tools and AI in health [11]. At an individual level, PROMs facilitate shared decision making, screen or monitor symptoms and provide timely care tailored to individual needs [11,12,19]. Meanwhile, however, it has become obvious that one single (as opposed to a multidimensional) PROM may not cover all aspects of health for a given disease at various timepoints. Further, the definition of which dimensions of disease are to be included is obviously critical. A real challenge to introduce PROMs is that today’s healthcare personnel, including clinicians, are not formally trained to consider also the impacts of social determinants of health (SDOH) on health outcomes [17,20] while delivering care [21,22,23]. According to the County Health Rankings Model, SDOH, such as health behaviors, socioeconomic factors and physical environment, contribute to 80% of the clinical outcomes in a community. In contrast, clinical care contributes to the remaining 20% of clinical outcomes [21,24,25]. Clinicians rely heavily on biomarkers and diagnostic test results to guide their decision-making. Shared-decision making preference elicitation and documentation remain challenges in today’s healthcare system, and preferences related to quality of life should be considered in treatment decision making [26]. To cover all healthcare dimensions, Khurana et al. developed a Whole Person Health Score (WPHS) that quantifies a person’s health into six domains: physical health, emotional health, resource utilization, socioeconomics, ownerships, and nutrition and lifestyle [21]. The WPHS extends the physical health assessment by five more dimensions to cover all possible parameters impacting the course of a disease. Ideally, these six dimensions are represented when a HRQoL instrument is developed. An instrument covering all health dimensions for a given disease including the incorporation of the patients’ view to define the shared value is of great importance to ultimately realize value-based healthcare (VBHC), which will be the base to create a sustainable healthcare system with cost control (Table 1). The question remains, however, how this is being realized specifically for sarcoma patients.

In this review, we first address the challenges of introducing PROMs in routine sarcoma patient care, to then, based on the literature, reason about the selection of which aspects need to be covered. In a further step, we describe a sarcoma-specific instrument which basically is composed of established PROMs. Last, we introduce the interoperable digital sarcoma platform which allows for simultaneous data assessment and its analysis.

## 2. Challenges to Introduce PROMs for Sarcoma Patients

Sarcomas represent an extremely heterogenous disease. While being rare or ultra-rare, they are composed of more than 175 distinct diagnostic entities, and occur at all possible anatomic sites of all age groups. Treatment is transdisciplinary, and while surgery is the mainstay of treatment, it is extremely complex because surgical techniques vary greatly from one anatomical site to others, and one surgeon nowadays is unable to cover the entire surgical spectrum of techniques at the required level [27]. Several disciplines together determine the successful outcome of the patients, and weekly multidisciplinary tumor board meetings have proven to be instrumental to achieve this [28,29,30,31,32]. While choosing a sarcoma instrument to define patient-defined quality of care, it cannot only focus on one single diagnostic or therapeutic aspect, but it must incorporate the sum of care provided by all disciplines because its success is determined by the sum of all treatments. As sarcoma patients need life-long follow-up for potential late effects associated with their disease, the assessment has to be designed longitudinally over the entire cycle of care, from the initial work-up of the patient, all types of treatment combinations, until last follow-up or death. If we want to assess the health of sarcoma patients using the WPHS framework, we need to assess a broad range of outcomes. There is already a multitude of PROMs for sarcoma patients available, both for assessing experience and outcomes of treatment [33,34,35,36,37,38,39,40]. Ideally, the information assessed through PROMs in sarcoma could be integrated in some overall sarcoma-specific instrument to define quality of care. The main prerequisites include the possibility of routine collection of data (as opposed to use in clinical trials only) as well as the allocation of PROMs that correspond to patients’ clinical metrics such as anatomic location, diagnostic subtypes, type of therapies, age and gender as well as prognosis [36,37]. It currently remains an open question whether generic or cancer-specific questionnaires will push through [41]. Generic questionnaires alone (such as EQ-5D) may not specifically enough address the needs for a given disease at any given timepoint of the care cycle such as sarcoma, while as opposed to a newly developed sarcoma-specific instrument, an established PRO may allow for cross-comparison among other diseases and benchmark comparisons to the standardized normal population. 

## 3. Which PROMs Are Being Used for Sarcoma Patients?

In a scoping review, Almeida et al. mapped the reported PROMs in sarcoma patients from the available literature and how they were measured, focusing specifically on the nature, extension and reach of research related to PRO and what instruments were being used to assess these [33]. They stressed the importance that PROMs include multidimensional assessments of quality of life, that the evaluation be longitudinal, and that anatomic location should be instrumental to include. Although the assessment of different time points of the care cycle—with respect to different diagnostic procedures or treatments—would provide important information, the complexity of sarcoma as a disease may hinder such introduction and utilization of PROMs with common approaches. Almeida et al. therefore concluded that there must be a new and sarcoma-specific measurement strategy to mirror the quality of sarcoma care [33]. 

Martins et al. reported on the sarcoma measure (SAM) [34]. They defined 22 items reflecting physical, emotional, financial well-being as well as sexuality and coined the term “sanxiety” of sarcoma patients. The SAM is a patient reported experience measure that can be used in clinical practice for all patients irrespective of age, type of sarcoma or treatment status. Some criticize that it is impossible to have one sarcoma-specific measure that meets the needs of clinical practice, academia and industry [41]. Others put fourth that SAM is only an experience but no outcome measure [36,37]. 

Within the spectrum of mesenchymal tumors, there are diagnosis specific PROMs for desmoid patients. In the Profiles study, Schut et al. created a disease-specific, desmoid-type fibromatosis questionnaire (DTF-QoL) covering 173 questions (which takes up to one hour for the patient to fill out) and compares it to the well-established and generic EORTC-QLQ and EQ-5D questionnaires, which are currently under investigation. They foresee to use it longitudinally over the entire care cycle depending on their prospective findings upon the conclusion of their study [35,42]. 

Den Hollander et al. performed an exhaustive systematic literature review unravelling the heterogeneity of disease and sarcoma patients’ health related quality of life [37], specifically focusing on the anatomic tumor location. They analyzed fifty-four different questionnaires, most often cancer-generic or generic HRQoL questionnaires. While they found that sarcoma patients in general reported lower HRQoL than the general population, they identified distinctive patterns with respect to symptoms, physical functioning, disability and psychosocial well-being depending on the tumor’s location. They also found that other factors such as disease stage should be taken into account to prioritize patients’ needs. These authors concluded that a sarcoma-specific strategy should be developed and used covering the heterogeneity of sarcoma, including anatomic location specific issues to improve personalized HRQoL assessment in clinical practice. As a follow-up on this, den Hollander et al. recently published a study protocol to develop a sarcoma specific instrument to develop a comprehensive list of HRQoL issues relevant to sarcoma patients, as well as a measurement strategy indicating which issues should be evaluated in certain subgroups [37]. While such an approach is extremely useful and will hopefully lead to customized measures, it will represent an entirely new instrument which cannot be cross-referenced with existing ones. Therefore, an alternative approach may include the introduction of a set of validated PROMs, which are already being used in many other cancer types and, importantly, for which there is most often information on the normal population available for comparison and benchmarking. These are designed to cover not only different dimensions of health, such as for example physical and mental health, but also the entire care cycle with customized measurements based on type of therapy performed and disease status. A sarcoma-specific instrument to cover the entire longitudinal care cycle using established PROMs would allow cross-referencing with patients with other cancers as well as with the normal population, thereby greatly enhancing the establishment of quality standards and ultimately sustainable cost accounting in sarcoma care.

## 4. Sarcoma-Specific HRQoL-Instrument Based on Generic PROMs 

The major challenge in defining a sarcoma-specific HRQoL instrument is the complexity of the disease itself and the respective multidisciplinary treatments at various timepoints, with greatly changing expectations from the patients’ side over time depending on the disease status. In designing the sarcoma-specific HRQoL instrument, we addressed the following challenges: (1.) use of generic and well-established PROMs to allow benchmarking with other diseases as well as the normal population; (2.) to cover the main WDPS aspects such as physical and emotional health, resource utilization (which is specifically important in sarcoma patients with respect to rehabilitation), socioeconomic aspects, ownership, nutrition and health; (3.) longitudinal assessment from first time presentation until last follow-up or death; (4.) individualized assessment by assigning only PROMs relevant for treatment received as well as follow-up status (Table 2). The herein presented sarcoma-specific instrument includes a variety of PROMs for the baseline visit of the patient. It includes the disease specific physical (EQ-5D-5L) as well as the overall health (PROMIS Global-10; WHO-ECOG) questionnaires. For example, indicated pain level (disease specific) may not necessarily be caused by the soft tissue tumor at the forearm itself but by unrelated back pain (overall health related), which must be distinguished in the assessment. Emotional as well as socioeconomic factors are pivotal and are covered using specifically EQ-VAS, Brief Symptom Inventory (BSI-18) and the work ability index (WAI). As the biopsy is an invasive procedure, it has an important bearing on the patient and therefore must be addressed separately as well. We are currently developing a specific mesenchymal tumor biopsy PROM (MTBP) including 10 questions and this will be reported separately. Toronto Extremity Salvage scores (TESS) as well as Musculoskeletal Tumor Society (MSTS) scores were specifically designed for the extremities and are globally accepted and used. The Transatlantic Australasian Retroperitoneal Sarcoma Working Group (TARPSWG) is in the process of establishing a specific PROM for visceral sarcoma surgeries and will be included here. With respect to radiation therapy, there are two PROMs introduced which evaluate patient-reported symptomatic toxicity (Patient-Reported Outcomes version of the Common Terminology Criteria for Adverse Events (PRO-CTCAE) as well as the local effects by the patient (Local effects of radiation therapy). Medical oncologists often use the widely accepted HRQoL measure EORTC-QLQ30 specifically for randomized controlled trials. It provides a holistic overview and includes other well-established PROMs such as MDASI. Obviously, once the patient has completed the therapy and undergoes regular follow-up visits, different needs have to be addressed such as rehabilitation capacities. We are currently working on a synthesis of PROMs which bases on the presented PROMs herein, but with adapted weighting to address specifically emotional health, socio-economic factors, ownership and resource utilization such as rehabilitation capacities and potential. 

This herein presented sarcoma-specific HRQoL instrument aims to address the specific needs and challenges of the sarcoma patient depending on disease status as well as type and time-point of therapy, or follow-up. This information will help to compare the different patients for a given time point or treatment type, but also to measure changes over time or effectiveness of new interventions for the individuum. Its design to assess the patient over the entire care cycle allows the personalized longitudinal assessment. Ideally, the patient receives the opportunity to monitor their own disease status and can objectively follow their own progress of disease development, which may enhance the transparent exchange with the physician. 

## 5. Interoperable Digital Platform: Data Assessment and Analysis

The large volume of data produced in healthcare overall and also proposed herein to be assessed with the PROMs presents a major obstacle and threat to routine practice [53]. As a consequence, there is a seemingly unsurmountable threshold to introduce a set of PROMs into routine medical practice, being used at best under standardized conditions such as in randomized controlled trials, and to assess these data several times for the same patients over time. Data management is a tremendous challenge, but the advent of digital transformation may lead to the disruption of current practices and will revolutionize healthcare in the decade to come. There is still an ongoing debate on whether PROMs are being assessed on paper versus electronically; although, it has been shown that the latter far exceeds the potential disadvantages, and manual analysis of paper PROMs is not affordable anymore given the current overall labor shortage [9,54,55,56]. 

**Figure 1:**
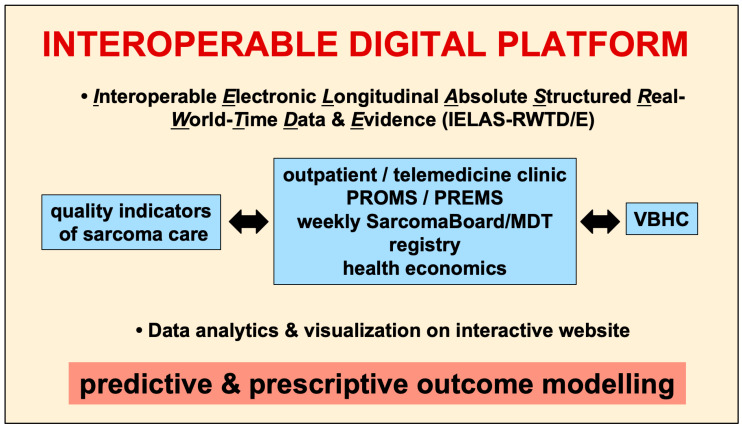
The interoperable digital platform integrates all available data of work-up, therapy and follow-up on the patient, based on quality indicators including the sarcoma-specific HRQOL instrument. It is the node of communication and exchange between patients and care providers of any level. It assesses and integrates routine, structured data in real-time of absolute and prospective patient numbers over time (IELAS-RWTD/E), which ultimately enables VBHC by attributing cost tags. The interoperable digital platform visualizes the descriptive data analytics on an interactive website, and ultimately allows predictive and prescriptive outcome modeling. PREMS: patient reported experience measures; MDT: multidisciplinary tumor board/Sarcoma Board.

Given the complexity of sarcoma as an extremely heterogenous disease with complex transdisciplinary treatment interactions, there is ideally an interoperable digital platform which integrates all diagnostic and treatment relevant aspects of sarcoma care (Figure 1). As such, the data generated from PROMs assessment can be analyzed in the context of all clinically relevant parameters such as disease status, exact pathological diagnosis, anatomic location as well as treatment decisions at the weekly multidisciplinary tumor board. Further, it also allows automatically tailoring (and therefore decreasing the time spent to fill out a questionnaire) the specific questions of a given PROM to the patient’s specific situation. For example, if the patient on the EQ-5D-5L has “no emotional constraints”, then it will not be necessary to fill out also the BSI-18 to assess the specifics of emotional constraints. As such, by individualizing the questionnaire to the patient’s need, it will be more attractive for the patient to spend more or less time to answer all the questions. Another advantage includes the generation of automatic alerts for the patient by the interoperable digital platform. Having integrated, for example, the date of surgery (or any other treatment aspect), the system can send an alert to the patient at predefined intervals to assess the respective PROM over time. The interoperable digital platform is able, based on the patients’ answers, to prepare or generate individualized reports for the regular clinical outpatient or telemedicine visits. We foresee that such an interoperable digital platform is able to integrate all patient related information (i.e., clinical, molecular, as well as economic parameters) as well as all stakeholders of patient care and to generate IELAS-RWTD/E [57,58]. Therefore, the implementation of PROMs has to be viewed as an integral part of patient care to generate robust data to optimize treatment decisions for the patient, but also to define quality of care, which ultimately is the prerequisite for value-based healthcare, paving the way to establishing a sustainable healthcare system. In a next step, together with all physician-based data of clinical metrics, such an interoperable digital platform allows the holistic analysis of all data dimension parameters, thereby allowing the generation of IELAS-RWTD evidence, which can be instantly analyzed and visualized on a protected interactive website. Ultimately, through machine learning algorithms, such a set-up allows predictive and prescriptive outcome analytics, which is the prerequisite for the upcoming precision medicine era (Figure 1).

## 6. Discussion

The patient’s perception on quality of care and treatment effectiveness are meanwhile established predictors of outcome. The introduction of a sarcoma-specific HRQoL instrument therefore is indispensable, specifically for a complex disease requiring the interplay of multiple disciplines. The literature suggests that novel questionnaires be designed to better reflect the heterogeneity of sarcoma as a disease as well as its treatment because one single PROM does not cover all required needs.

Herein, an alternative approach to define a sarcoma-specific HRQoL instrument is presented using a sum of established generic PROMs which are assessed depending on the respective status of the longitudinal care cycle of the patient [8]. This approach allows comparison and benchmarking with other diseases and, importantly, with the normal population [59]. This is in contrast to the suggestion by Den Hollander et al. who develop an entirely novel sarcoma-specific questionnaire [37]. It will be very interesting to see which parameters this group will ultimately include in their questionnaire, and how it will compare to the approach presented herein. Obviously, the implementation of this sarcoma-specific HRQoL faces some challenges. Both physicians and patients have to be trained for a novel ecosystem of data handling and assessment by on-site education during their outpatient visit. Similar to an iPhone use, the ease of self-explanatory handling of the program to enter the data does not really present a hurdle. Each patient (or telemedicine visit via a link) receives an iPad to enter the data and, if needed, receives instant support for program handling. Data themselves are stored and protected regarding ethical and legal issues, and only deanonymized data are used for exchange in the context of national and international comparisons, which is very important to scale globally and is also shown by others [60]. To achieve this, we have created the Sarcoma Academy to foster quality in sarcoma care by organizing monthly webinars (www.sarcoma.academy; accessed on 1 March 2023). The patient and their treating physician have access to their own data and represent the main contributors and also the main stakeholders. Once predictive modeling is realized, the results shall benefit the patient and his treatment.

In designing a sarcoma-specific HRQoL instrument, a holistic inclusion of all possible health dimensions is preferred. Ideally, it includes the entire longitudinal care cycle of routine care with the data dimensions as defined by WPHS, structured data which are instantly available for both patients and care providers, to ultimately achieve IELAS-RWTD/E analytics and precision outcome modeling [61]. Obviously, such a comprehensive amount of data cannot be handled with common approaches; a new ecosystem of data management is therefore required in healthcare. With the exciting opportunities created by digital transformation, the definition of different types of data will become increasingly important in the future. The interoperable digital platform is designed as a node for the integration of data generated from both the care providers, as well as the patients, to internationally exchange and scale to create evidence through analytics and, ultimately, knowledge. For this purpose, the interoperable digital platform presented herein allows *E*lectronic capturing of data which are *L*ongitudinal and prospective if they cover the entire care cycle, and *A*bsolute if they are all consecutive and not just a selection of patients is included. The assessment has to be designed so that all data will be *S*tructured instead of unstructured. *R*eal-*W*orld data refers to routine data assessment, and real-*T*ime refers to the instant availability of these *D*ata. The interoperable digital platform allows an automated analysis of these data, thereby creating *E*vidence. As such, it is designed to include all data dimensions as represented by IELAS-RWTD/E. Integrating all data dimensions of quality indicators of care from both the patients’ and physicians’ view sets the stage for an integral evidence analytics. Additionally, this in turn is the prerequisite for predictive modeling to individualize therapy in the future, thereby realizing precision care. Integrating the sarcoma-specific HRQoL instrument into an interoperable digital platform allows the analysis of each single parameter of the HRQoL instrument with clinical metrics such as specific type of diagnosis, anatomic localization and therapy aspects. It facilitates the coordinated exchange of information between patients and care providers to be transparently shared by both. The interoperable digital platform therefore provides not only information and knowledge for the physician but also for the patient. Such an interoperable digital platform can also be designed to be an integral part of an institutional electronic health record (EHR) system. It is foreseen that an EHR will be composed of many other disease specific interoperable digital platforms, as presented herein for sarcoma. In its entirety, such EHR is then able to not only provide information on the entirety of all medical care provided over the care cycle, but it also assesses the treatment effectiveness both from the physicians’ and patients’ perspectives with transparent real-time analytics. Further, attributing now a cost tag to each quantifiable structured data unit, the individual costs of treatment for a given disease can be determined longitudinally. This in turn allows the definition of shared value (which equals quality and outcome over costs of the entire longitudinal care cycle), as Porter et al. defined the concept of value-based healthcare [3,5]. 

In summary, a holistic approach in designing a sarcoma-specific HRQoL instrument includes the sum of multiple generic PROMs tailored to the specific steps of the entire care cycle. While this, on the one hand, allows for comparison and benchmarking with other diseases as well as the normal population, the large data volume cannot be handled with standard approaches. A novel interoperable digital platform not only integrates both patient- and physician-based quality indicators but also allows IELAS-RWTD/E analytics, paving the way to precision medicine and value-based healthcare [62]. The holistic assessment of the health of sarcoma patients and survivors will contribute to tailoring care to patients’ needs and, ultimately, improve health. Further, such an approach will be indispensable to associate treatment effectiveness with healthcare cost control, which is the prerequisite for a sustainable healthcare system.

## Figures and Tables

**Table 1:** Summary of main challenges, bibliography included in this introduction and its added value.

Challenge	References	Added Value
Where do we want to go?	[8]	This article describes the transition from the current care to the future state of how our healthcare system will look like in 2030, specifically emphasizing the potential of digital transformation.
What is good health?	[18,21,22,23,24,25,26]	These articles define the social determinants of health and the delivery of care. Health behaviors, socioeconomic factors and physical environment contribute 80% to health outcomes, whereas clinical care only contributes the remaining 20% to clinical outcomes. For these reasons, the Whole Person Health Score was created.
What is the current problem?	[1,2,6,7,27]	Healthcare costs represent a seemingly unsurmountable problem, and lead us towards an unsustainable trend. While perceptions of value differ among patients and providers, shared decision-making regarding data assessment and documentation remain challenges specifically because most of the healthcare data are unstructured und therefore not ready for analysis.
Potential solutions?	[3,4,5]	Improving value benefits may lead to a sustainable health system. A novel ecosystem centers on the shared value being the multidimensional relationship between health outcomes and costs incurred to deliver these, as defined by the value-based healthcare principle. For these reasons, quality of care must be defined and assessed using structured data, to which then cost tags can be attributed which allows the definition of the entire costs of a given diagnosis over the entire care cycle.
Why are PROMS important?	[9,10,11,12,13,14,15,16,17,18,19]	These articles summarize how PROMS assess treatment effectiveness and outcome, and show that they can improve survival. PROMS have to be designed such that they cover the entire spectrum of the social determinants of health as suggested by WPHS but are nevertheless disease-specific.

**Table 2:** Sarcoma-specific HRQOL instrument: summary of established PROMs assigned at the different timepoints and for different treatment status.

Time Point of Assessment & Therapy Status	Type of PROM
Work-up at diagnosis Regular visits during therapy	WHO-ECOGPROMIS [43]
	EQ-VAS [44]
	EQ-5D-5L [45] BSI-18 [46] WAI [47]
Biopsy	Mesenchymal Tumor biopsy PROM *
	(MTBP)
Surgery	TESS (upper/lower extremity) [48]
	MSTS (upper/lower extremity) [49]
	Visceral (TARPSWG) *
Radiation Therapy	Local effects of therapy
	PRO-CTCAE
Chemotherapy	EORTC-QLQ-C30 [50,51]
Follow-up visits after completion of therapy	Combination of above

* in development; ROMS are assessed at each follow-up visit as suggested by Wilson et al. [52], Abbreviations: WHO-ECOG: World Health Organization–Eastern-Cooperative Oncology Group; PROMIS: Patient reported outcome measurement information system; EQ-VAS: EuroQuol Group visual analogue scale; BSI-18: Brief symptom inventory; TESS: Toronto extremity salvage score; MSTS: Musculoskeletal tumor society; TARPSWG: Transatlantic retroperitoneal sarcoma working group; PRO-CTCAE: Patient-reported outcomes Common terminology Criteria for adverse events; EORTC-QLQ-C30: European Organisation for Research and Treatment of Cancer—Core quality of life questionnaire.

## Data Availability

Not applicable.
